# Depression and Its Associated Factors Among the Elderly Population in India: A Review

**DOI:** 10.7759/cureus.41013

**Published:** 2023-06-27

**Authors:** Deepak Vishwakarma, Abhay Gaidhane, Sudha R Bhoi

**Affiliations:** 1 School of Epidemiology and Public Health, Datta Meghe Institute of Higher Education and Research, Wardha, IND; 2 Department of Community Medicine and Public Health, Datta Meghe Institute of Higher Education and Research, Wardha, IND

**Keywords:** old age, older people, mental health, geriatric, depression

## Abstract

Over the last few decades, life expectancy has increased, particularly for old age people. This has resulted in an increased number of family members at a given time leading to more crowded households and thus causing stress in members of joint or large families. Lack of family support, the death of a loved one, isolation in the community due to poor physical health, and generational and communication gaps within the family, even though they reside under the same roof, are just a few of the things that can cause loneliness. These issues affect the mental health of elderly persons and may occasionally result in depression. Depression's high morbidity and mortality rates, particularly in older people, make it a serious public health concern. This review summarises that elderly persons have a higher prevalence of depression; regarding routine depression screening for the older population and their counselling, no precise guidelines are available. We used Medical Subject Heading (MeSH) phrases to search for published articles/studies/research in the English language in PubMed, Scopus, and Google Scholar. We also searched numerous government websites for recent data on geriatric depression and we analysed 35 articles. Old age is the transition stage where an individual must deal with various physical and mental health problems due to brain ageing that leads to changes in behaviour that affect their social well-being. The existing mental health programme should pay more attention to the problems with senior depression. In order to deal with the problem of depression, they might also involve non-governmental organisations (NGOs).

## Introduction and background

Depression is a serious mental health issue that poses a significant public health concern. In India, senior citizen comprises 10.1% of the total population [1]. And if they go undetected, depression in the senior population can be projected to contribute significantly to the disease burden [2]. The global population is rapidly getting older. Between 2015 and 2050, the proportion of older people is expected to double, from 12% to 22% nearly. In absolute terms, it is predicted that the number of people over 60 years will increase between 900 million to 2 billion [3]. Older individuals experience distinct mental and physical issues that must be addressed [3]. A psychological or neurological disorder affects more than 20% of adults aged 60 years and older (headache disorders not included), and 6.6% of all disabilities (DALYs or disability-adjusted life years) in people over 60 years are caused by mental or neurological disorders [3]. According to the Indian government's National Policy on Older Persons, a senior citizen is 60 years of age or older [4]. In 2011, India had 98 million senior persons, predicted to rise to 143 million by 2021, with females accounting for 51% [4]. The typical Indian's life expectancy has increased from 64.6 years in 2002 to 70.19 years in 2022 [1]. By 2050, the proportion of Indians aged 60 or over will reach 19.5% of the country's total population, which is concerning and warrants prompt intervention [1].

Depression is a common mental disorder that affects 20% of the geriatric population and is characterised by a gloomy feeling, a loss of enjoyment and interest, lower energy, guilt or a lack of self-worth, disrupted sleep or meals, and impaired concentration [4]. The frequency of chronic non-communicable diseases is increasing along with the ageing population and consequently, it is likely that depression would become more frequent [5]. The rise of nuclear families has raised the expense of living, and changes in family priorities have harmed India's elderly. Old age, deterioration of physical health, death of a spouse, dependence, helplessness, and poor self-esteem are risk elements that affect the severity, prevalence, and quality of life of mental illness [6]. Depression reduces one's life quality and increases one's dependence on others. Due to their declining health conditions, older people become dependent on others for their day-to-day activities which creates stress among them [5]. Depression is a serious public health problem since it substantially contributes to suicide [7]. In various studies, we discovered that older people having depression have more co-morbid conditions than those without depression.

This review aims to highlight the prevalence of depression in elderly persons. Regarding routine depression screening for the older population and their counselling, there are no precise guidelines available. In addition to the shortage of counsellors or psychiatrists in the villages, social taboos may also be a factor. Lack of family and societal support for older people, especially, over 60 years could be another factor. The present review was conducted to inform policymakers about the burden of geriatric depression so that they can take appropriate measures to deal with it.

## Review

Methodology

**Figure 1:**
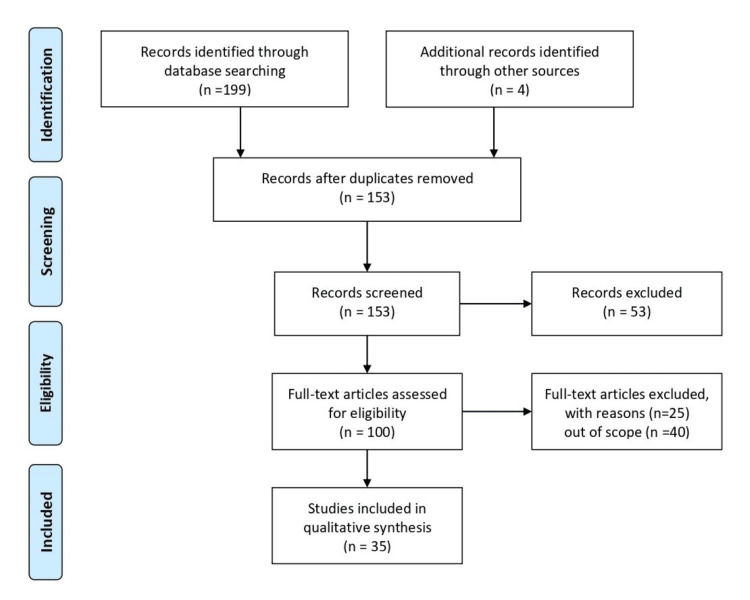
PRISMA flow diagram depicting how the studies were selected PRISMA: Preferred Reporting Items for Systematic Reviews and Meta-Analyses

We used Medical Subject Heading (MeSH) phrases to search for published articles/studies/research in the English language in PubMed, Scopus, and Google Scholar, including ''Depression” “Geriatric" "Elderly people” "Mental health" “Risk factors” and Boolean operators “AND/OR”. We included various studies on depression among older people and its risk factors. The Inclusion criteria followed articles ranging from 1987 to 2023 that focus solely on depression among older people and its risk factors. Figure 1 below depicts how the studies were selected.

Discussion

India is a country where joint family is the established norm. Everyone shares duties, possibilities for financial gain, social obligations, etc., in these households, and younger siblings were highly regarded. The speed of economic and technical development over the past 50 years has made it necessary for society to adapt to rapid industrialisation and technological developments in information and communication. Joint family structures have become fragmented due to rapid population migration from ancient homelands-off places in search of financial gains. National programmes for population control also reduce the number of households. Children are also moving for jobs and education. As a result, the number of nuclear families is rapidly increasing. Older people are abandoned in their ancestral homes with little physical, psychological, emotional, financial, or social care [8,9]. The process of shifting family structures in Indian villages, youth movement from rural to urban areas, and the breakdown of the rural fabric of society are having an adverse impact on the elderly living in rural areas. They are more vulnerable since they do not have the benefit of accessing social assistance and medical facilities [10]. Families have an essential role in the lives of the elderly, particularly in Asian cultures where the joint family system predominates. Even though quickly changing urbanisation impacts structures, families still play a crucial role in providing security and support for the elderly in nations like India. In a family, older people have a position of authority that calls for a great deal of respect and admiration. The elderly rely on their partner in addition to their children, mainly their male children, as girls typically move out of their parent's homes once they get married [11,12].

An underappreciated issue in India is depression among elderly people. Like other regions of the world, India has seen an increase in the number of elderly people as a result of longer life expectancies, a switch in illness patterns from communicable to noncommunicable, and lower fertility rates. Due to industrialization, individuals today choose to live in nuclear families, both in rural and urban regions. This leads to loneliness among the elderly and a lack of social and familial support, which worsens their already precarious health conditions and ultimately makes them vulnerable to depression. Even though this situation is not new, depression among the elderly population continues to be hidden and is receiving little attention [13,14]. In terms of improving elderly people's quality of life and thus prolonging their life expectancy, primary care physicians and family medicine specialists are crucial. Family medicine, as is well known, employs a holistic approach that addresses the patient's views, worries, and expectations as well as the medical treatment of the disease. It may also involve family members or other carers, depending on the circumstances. That highlights the need for primary care physicians to concentrate on depression, a mental health issue frequently present in older patients. A family doctor can help maintain and improve family connections by counselling and inspiring those caring for the elderly. This is based on the risk factors that have been found [15,16].

Obesity and its problems are more likely among elderly persons from middle and upper-income groups due to sedentary lifestyles and decreased physical activity, which makes them dependent on other family members. As a result, older people are more likely to be abused by family members and in institutions. This encompasses physical abuse (causing discomfort or harm), psychological abuse (causing mental distress), and criminal exploitation [13]. There are several risk factors for depression in older individuals, which are listed below in Table 1.

**Table 1:** Factors associated with depression Data sources [4,14-20].

S.No.	Factors	Description
1	Age	Age is one of the most significant non-modifiable risk factors for mental disease; therefore, health services should consider that. As people reach the age of 60 and beyond, they are more likely to have substance abuse disorders, mental illnesses, neurological conditions, and other health issues, including osteoarthritis, hearing loss, and diabetes [4,14].
2	Gender	Women are more prone than men to suffer from severe depression due to the transition to menopause, a condition known as early menopause, where hormone levels may fluctuate wildly. When estragon levels are dramatically lowered, as they are during early menopause and following menopause, chances of depression may also increase. Most women who experience bothersome symptoms of menopause do not go on to experience depression. However, the following elements may increase the risk of poor or interrupted sleep, A history of anxiety and depression, stressful situations, and a rise in body mass index or weight (BMI) [15].
3	Low income	The inability of elderly persons to support themselves due to physical infirmity, retirement from employment, or low-income levels that force them to depend on their offspring for subsistence is another factor contributing to depression in this population.
4	Single/Widowhood	Widowhood, divorce, and separation are more common in women than in men, and thus those who have experienced a severance have much more depressive symptoms than those who are married [16]. Due to being single, their social circle has altered, and they have become more socially isolated, which has increased their depression [17].
5	Unemployment	In most countries, employment is one of the indicators used to assess the country's economic and social situation. Numerous studies have been conducted on the connection between depressive symptoms and unemployment. According to studies, unemployment can result in diminished hope and financial difficulties, increasing depression [18].
6	Illiteracy	A person with a low degree of education finds it challenging to adequately complete particular duties in an urban city. Doctor consultations, completing paperwork in English, and handling household finances are a few examples. Older people who have these issues at a higher chance of developing depression [19].
7	Nuclear family	Urban housing shortages, which prevent large families from sharing a home, and employment options, which force the younger generation to undergo economic production separation, are likely to blame for the dissolution of the nuclear family system. One key result of this separation seems to be the loss of "elderly control" over the younger generation. As a result, family systems are more likely to form because the older man no longer controls, the younger generation through ownership of the family's wealth. Elderly co-residence with adult children declines as a result of nucleation, which also results in a decline in the elderly's care and assistance [19,20].
8	Poor physical health	Poor physical health increases the likelihood of diabetes and high blood pressure, which increases the chance of stroke, heart disease, and other senile diseases.

The senior population in India and Asia is growing, suggesting a need to expand the field of Geriatric Psychiatry. And also need to know the commonly occurring disorders in this population [21,22]. The 2010 National Program for Elder Health Care (NPHCE) addresses the numerous health problems of older people. Nevertheless, the integrated approach could be better due to a lack of infrastructure and educated staff specifically trained to address the mental health problem of old age [23,24]. In collaboration with the NPHCE, the mental health programme can strive to improve the healthcare system so that earlier detection and evidence-based mental health treatment concerns in the elderly [25]. The Government of India has launched several measures to improve the health of older people. Among these are the 2007 Maintenance and Welfare of Parents and Senior Citizens Act and the National Policy on Older Persons. The National Programme for Health Care of the Senior, a relatively new initiative focused on the elderly, was launched by combining these two strategies. The advantages include free and exclusive senior-only medical facilities provided by the public health delivery system. Preventive, promotional, and curative treatment and referral services are all included in the NPHCE health packages [26]. The packages are dispersed according to the health facilities, which are sub-centres, primary health centres, community health centres, and district hospitals. The regional centres now have geriatric clinics and wards [27]. However, this does not address the need for older adults to interact with family and other people to deal with mental health issues. Although the Parents and Senior Citizens Act 2007 is in place, still older people are being abandoned, so strict and mandatory sentences should be passed for those who abuse and abandon older people. Numerous research studies support the percentage of senior Indians who suffer from depression, listed below in Table 2.

**Table 2:** Studies evaluating the prevalence of depression in elderly people PHC- Primary health care; GDS- Geriatric depression scale; ICD-10- International classification of diseases, tenth revision Data source [1,4,6,7,27-35].

Author	Place	Cut-off age (in years) for defining elderly	Sample recruitment	Diagnosis of depression based on	Prevalence in (%)
				Community-based studies	
Naik and Nirgude [1]	The rural community of South India	60-70	230 community-based systematic random sampling	GDS-30	79.5% had mild depression, 20.4% had severe depression
Bincy et al. [7]	The rural area of Tamil Nadu	60-69	2400 community-based stratified random sampling	GDS-15 (short version)	67.5% prevalence of depression is determined
Laksham et al. [28]	Village area of Puducherry	60 and above	359 community-based systemic random sampling	GDS-15 (short version)	56% of people reported having mild depression. 25% of people reported moderate depression. 19% were severely depressed.
Goswami et al. [27]	The rural area of Maharashtra	60-70	287 community-based random sampling	GDS-30	26.72% had mild depression. 15.17% had severe depression.
Mandolikar et al. [4]	Urban community Karnataka	60-64	229 – community-based	GDS-30	75.5% had severe depression and 84.97% had mild depression.
Sahni et al. [29]	Kirpind village north India	60 and above	183 community-based house-to-house visits.	GDS-15 (short version) Hindi language	59.3% were not depressed. 33.9% of people had minimal to moderate depression. 6.8% had a major depressive disorder.
Thilak et al. [30]	The rural area of Kannur North Kerala	60 and above	250 community-based convenient sampling	GDS-15 (short form)	72.4% had depression
Dhar et al. [31]	Mustafanager of Devangere City Uttar Pradesh	60 and above	350 community-based simple random sampling	GDS-30	33 % of people had only minor depression. 6 % had severe depression.
Goyal and Kajal [32]	The southern part of Punjab	60 and above	100 community based	GDS-30	60% had mild depression. 17% had severe depression.
Reddy et al. [6]	The rural area of Tamil Nadu	60	800 community based	GDS-15	43.25% were depressed
Barua and Kar [33]	Three talukas of the Udupi district of south India	60	627 community-based simple random sampling	WHO (five) Well-being Index (1998, version) and (ICD-10) depression inventory	21.7% were determined
Rathod et al. [34]	The rural area of Aurangabad Maharashtra	60 years and above	400 house-to-house survey, a systemic random sampling	Major depression inventory (MDI) scale	16.75% were determined
Sanjay et al. [35]	Three (PHCs) under the field practice area of a private medical college in Bengaluru	60 years and above	992 community-based random sampling	Geriatric depression scale (GDS-15)	38.1% were determined

## Conclusions

Older adults make significant contributions to our society as family members, volunteers, and consultants. Age, economic dependence, co-morbid conditions, and dependence on others for their routine activities are the key factors that raise a person's risk of getting depression. Older people can become more independent in daily tasks and less depressed by improving their general health. Additionally, financial support in the form of a pension can lessen older people’s sense of helplessness and enhance their mental health. Although several schemes are available for providing financial assistance to the elderly, because the standards for receiving economic benefits from the government are so difficult, they are not being utilised as they should be. To address the substantial societal issue of depression amongst the older population, screening services and appropriate psychological counselling must be accessible in the neighbourhood. Social security legislation must be modified to remedy this issue, and actions must be taken to promote community involvement. In this way, even if the younger children in the family move out, leaving the elderly parents alone, they can still contribute to strengthening the family's support system for them. The existing mental health programme should pay more attention to the problems of depression in senior citizens. In order to deal with the problem of depression, they might also involve non-governmental organisations (NGOs).
